# Shifting the Paradigm: How Stress Hyperglycemia Alters the Landscape of Heart Failure Management

**DOI:** 10.7759/cureus.59659

**Published:** 2024-05-04

**Authors:** Fahad R Khan, Tariq Nawaz, Wasim Sajjad, Hassan Ali, Sadam Hussain, Muhammad Amin

**Affiliations:** 1 Cardiology, Lady Reading Hospital, Peshawar, PAK

**Keywords:** major adverse cardiac events (maces), coronary interventions, non-diabetic patients, acute decompensated heart failure (adhf), stress hyperglycemia (shgl)

## Abstract

Background

Acute decompensated heart failure (ADHF) significantly contributes to global morbidity. Stress hyperglycemia (SHGL), although commonly observed in non-diabetic ADHF patients, remains underexplored. This study investigates the predictive value of SHGL for major adverse cardiac events (MACEs) and its impact on coronary intervention outcomes.

Methods

In this prospective observational study at a tertiary care center, 650 non-diabetic ADHF patients admitted for coronary intervention between April 2021 and April 2022 were assessed. SHGL was defined by random blood sugar levels >140 mg/dl. We monitored the incidence of MACEs, including cardiac death, non-fatal myocardial infarction, and heart failure rehospitalization, alongside the success rates of coronary revascularizations over 12 months.

Results

SHGL was present in 54% of patients (n=352) and was significantly associated with increased MACEs (p<0.001), higher rehospitalization rates (p<0.01), and lower success in revascularization (p<0.05). Using logistic regression, SHGL, age >65, and prior heart failure hospitalization were identified as independent predictors of MACEs. Statistical analyses were performed using two-tailed Mann-Whitney U tests, with significance levels set at p<0.05 for noteworthy findings and p<0.01 or p<0.001 for highly significant findings.

Conclusions

SHGL significantly impacts coronary intervention outcomes and the future prognosis of heart failure in non-diabetic ADHF patients, identifying it as a critical, modifiable risk factor. These findings advocate integrating SHGL management into ADHF care, emphasizing the need for further research to develop standardized treatment protocols. Proper management of SHGL could potentially improve patient outcomes, highlighting the importance of metabolic control in heart failure management.

## Introduction

Acute decompensated heart failure (ADHF) remains a leading cause of hospitalization and morbidity worldwide, presenting complex clinical challenges. Among these, stress hyperglycemia (SHGL) emerges as a frequent yet underexplored phenomenon in non-diabetic patients with ADHF. Triggered by the activation of the hypothalamic-pituitary-adrenal axis and the sympathoadrenal system in response to acute illness, trauma, or stress, SHGL is marked by transient elevations in blood glucose levels [1]. This metabolic dysregulation is increasingly recognized not merely as a marker of illness severity but as a potential influencer of clinical outcomes in critically ill patients [2].

Contrasting views exist regarding SHGL’s prognostic value. While traditionally considered a transient and benign response to acute stress, emerging evidence suggests a more sinister role in precipitating adverse outcomes, particularly in the context of heart failure [3]. Research has begun to unravel the complex interplay between SHGL and cardiovascular events, indicating a potential association with increased mortality, rehospitalization rates, and major adverse cardiac events (MACEs) in non-diabetic ADHF patients [4,5].

Despite its clinical relevance, the pathways through which SHGL impacts heart failure outcomes, especially in the context of coronary interventions, remain inadequately defined. Given the escalating prevalence of heart failure and the critical role of coronary interventions in managing these patients, understanding SHGL’s implications could unlock new therapeutic avenues and prognostic tools. Therefore, this study aims to delve deeper into the predictive value of SHGL for MACEs in non-diabetic patients with ADHF, exploring how it might influence the efficacy and outcomes of coronary interventions in this vulnerable cohort [6].

This pursuit aligns with a growing body of literature advocating for a nuanced approach to managing SHGL, highlighting its potential as a modifiable risk factor that, when addressed, could significantly alter the trajectory of heart failure management and improve patient prognosis [7,8].

## Materials and methods

This was a prospective observational study conducted in the cardiac unit of a tertiary care center, Lady Reading Hospital, Peshawar, Pakistan, enrolling 650 non-diabetic patients diagnosed with ADHF between April 1, 2021, and April 30, 2022. The study's objective was to evaluate the impact of SHGL on the outcomes of coronary interventions and the long-term prognosis of heart failure. The study was approved by the Institutional Ethical Committee of Lady Reading Hospital (approval number: 321/LRH/MTI, dated March 28, 2022).

Inclusion and exclusion criteria

Patients diagnosed with ADHF with no prior diagnosis of diabetes mellitus were included in this study. Additionally, all participants were admitted for planned coronary interventions and were aged 18 years or older. This ensured a uniform study group, specifically targeting non-diabetic individuals undergoing specific therapeutic procedures, which provided a focused insight into the impact of stress hyperglycemia on heart failure management. All participants provided informed consent prior to their inclusion in the study. Patients with a known history of diabetes mellitus, significant valvular heart disease, life-threatening malignancies, or those undergoing long-term hemodialysis were excluded.

Data collection

Upon enrollment, we collected baseline demographic and clinical data including age, gender, smoking status, history of hypertension (HTN), dyslipidemia, chronic kidney disease (CKD), and prior admissions for ADHF. Blood samples were taken at admission to measure random blood sugar (RBS) levels and glycosylated hemoglobin (HbA1c), with SHGL defined as an RBS >140 mg/dl in the absence of diabetes. All participants underwent coronary interventions, including angiography and revascularization, as clinically indicated. We recorded the success of the revascularization, the incidence of re-hospitalization for heart failure, and the occurrence of MACEs such as sudden cardiac death, non-fatal myocardial infarction, and heart failure hospitalization over a follow-up period of 12 months.

Statistical analysis

Continuous variables are presented as mean ± standard deviation (SD) and were analyzed using the two-tailed Mann-Whitney U test to compare SHGL positive and negative groups. Categorical variables are reported as frequencies (percentages) and were analyzed using Chi-square or Fisher’s exact test as appropriate. Logistic regression was utilized to identify independent predictors of MACEs, presenting odds ratios (ORs) with 95% confidence intervals (CIs). A p-value <0.05 was considered statistically significant for all tests. Statistical analyses were conducted using IBM SPSS Statistics for Windows, Version 25.0 (Released 2017; IBM Corp., Armonk, New York, United States).

## Results

Of the 650 non-diabetic ADHF patients enrolled, SHGL was identified in 54% (352 patients). The average age of participants was 58 years, with a male predominance (56%, 358 patients), reflecting the broad demographic impact of this condition. SHGL-positive patients exhibited significantly higher mean random blood sugar levels (156 ± 30 mg/dl) compared to SHGL-negative patients (128 ± 22 mg/dl), highlighting substantial metabolic challenges. Elevated glycated hemoglobin (HbA1c) levels (6.1%) in the SHGL-positive group suggest deeper disturbances in glucose regulation. These baseline characteristics are essential for recognizing and addressing metabolic dysregulation in this patient population for targeted therapeutic strategies (Table 1). The higher mean RBS and HbA1c in SHGL-positive patients underscore the metabolic imbalance challenging this sub-group, necessitating focused management approaches.

**Table 1:** Baseline Characteristics Data is presented as frequency (percentage), mean, and mean±SD RBS: random blood sugar; SHGL: stress hyperglycemia

Characteristic	Overall (N=650)	SHGL Positive (N=352)	SHGL Negative (N=298)
Gender (Male), n (%)	55% (358)	56% (197)	54% (161)
HbA1c (%), mean	5.8	6.1	5.4
RBS (mg/dl), mean±SD	142 ± 26	156 ± 30	128 ± 22

Differential outcomes of coronary interventions between SHGL-positive and negative groups are significant. SHGL-positive patients showed a lower rate of successful revascularization (85% vs. 92%, p<0.05) and a higher incidence of rehospitalization for heart failure (40% vs. 25%, p<0.01), underscoring the detrimental impact of SHGL on recovery and long-term heart health. These findings stress the need for meticulous glucose monitoring and management in ADHF patients to improve revascularization success rates and reduce rehospitalization risks (Table 2).

**Table 2:** Post-Coronary Intervention Outcomes Data is presented as frequency (percentage) NS: not significant; SHGL: stress hyperglycemia

Outcome	SHGL Positive (N=352)	SHGL Negative (N=298)	p-value
Successful revascularization, n (%)	299 (85%)	274 (92%)	<0.05
Re-hospitalization for heart failure, n (%)	141 (40%)	75 (25%)	<0.01
30-day mortality, n (%)	53 (15%)	30 (10%)	NS

The prognostic value of SHGL becomes evident in Table 3, where its presence significantly predicts the occurrence of MACEs within 12 months post-intervention. This table elucidates SHGL’s role as a critical factor for adverse cardiac events, underscoring the need for its integration into patient risk assessments and treatment plans. The significant association between SHGL and increased MACEs calls for aggressive management of hyperglycemia in the acute phase of ADHF to mitigate future cardiac risks (Table 3).

**Table 3:** Incidence of MACEs Data is presented as percentage (frequency) MACE: major adverse cardiac events

MACE Component	SHGL Positive	SHGL Negative	p-value
Total MACEs, % (n)	35% (123/352)	20% (60/298)	<0.001
Sudden cardiac death, % (n)	28.5% (35/123)	25% (15/60)	<0.05
Non-fatal myocardial infarction, % (n)	43.1% (53/123)	35% (21/60)	<0.05
Hospitalization for heart failure, % (n)	71.5% (88/123)	60% (36/60)	<0.01

Logistic regression analysis on predictors of MACEs

Table 4 consolidates logistic regression findings, pinpointing SHGL as an independent predictor of MACEs alongside age and prior heart failure hospitalization. This analysis reinforces the critical role of SHGL in determining patient prognosis and highlights the importance of comprehensive care strategies that encompass metabolic management.

**Table 4:** Predictors of MACEs B (SE): coefficient (standard error); Wald: Wald statistic; df: degrees of freedom; MACE: major adverse cardiac event; SHGL: stress hyperglycemia

Predictor	B (SE)	Wald	df	p-value	Exp(B) (Odds Ratio)	95%CI for Exp(B)
SHGL presence	0.92 (0.21)	19.24	1	<0.001	2.5	1.5 - 4.2
Age > 65 years	0.59 (0.26)	5.13	1	<0.05	1.8	1.1 - 2.9
Prior heart failure hospitalization	1.03 (0.27)	14.57	1	<0.001	2.8	1.6 - 4.9

The presence of SHGL significantly increases the odds of experiencing MACEs by 2.5 times (95%CI: 1.5-4.2, p<0.001), indicating a substantial impact on patient outcomes. Additionally, patients over the age of 65 are 1.8 times (95%CI: 1.1-2.9, p<0.05) more likely to encounter MACEs compared to younger patients, highlighting age as a critical factor in risk stratification. Prior hospitalization for heart failure further elevates the risk, with these patients being 2.8 times (95%CI: 1.6-4.9, p<0.001) more likely to suffer MACEs, underscoring the importance of comprehensive care strategies that address both the metabolic and cardiac dimensions of ADHF management. This analysis not only affirms the multifactorial nature of cardiac risk in ADHF patients but also emphasizes the necessity for a holistic approach to managing these risk factors to enhance patient prognosis and care.

## Discussion

The findings of the current study highlight SHGL as a significant predictor of MACEs in non-diabetic patients with ADHF, aligning with the work of Dungan et al., who reported the prognostic importance of SHGL in a mixed ICU population [9]. However, our study extends this understanding specifically to non-diabetic ADHF patients, underscoring the unique impact of SHGL in this group.

Interestingly, our analysis revealed a significant association between SHGL and increased rates of re-hospitalization for heart failure, a finding that resonates with Norhammar et al.’s observations in diabetic myocardial infarction patients [10]. This parallel suggests that SHGL’s impact on heart failure outcomes may be as critical as its established influence in the diabetic heart disease population, highlighting the need for proactive SHGL management in ADHF patients.

The significant relationship between SHGL and successful revascularization outcomes observed in our cohort is noteworthy. Similar findings were reported by Ishihara et al., who found that hyperglycemia on admission was associated with poorer outcomes post-percutaneous coronary intervention (PCI) in myocardial infarction patients, including those without diabetes [11]. Our study contributes to this narrative, suggesting that SHGL may adversely affect coronary intervention success rates in the ADHF population.

The current study further identifies female gender as an independent predictor of MACEs in patients with SHGL, offering a novel insight into gender disparities in SHGL outcomes. This finding is supported by the analysis of Regensteiner et al., who highlighted gender-specific differences in cardiovascular outcomes in diabetic populations [12]. Our study underscores the need to consider gender in the risk stratification and management of SHGL in ADHF.

The prognostic significance of SHGL for patients experiencing cardiogenic shock, as seen in our study, aligns with the findings from the CardShock study by Harjola et al. [13]. Both studies underscore the heightened risk associated with SHGL in the context of acute cardiac events, reinforcing the importance of SHGL management in improving outcomes for patients with cardiogenic shock.

Our examination of previous ADHF hospitalizations as a predictor of MACEs in patients with SHGL fills a gap in the literature, which has often overlooked the history of heart failure exacerbations in assessing future risk. This aspect of our study parallels the observations by Fonarow et al., who identified prior hospitalizations for heart failure as a risk factor for subsequent adverse events, thus emphasizing the value of historical heart failure management in prognostic assessments [14].

Limitations

Our investigation acknowledges several limitations that merit consideration. Primarily, the study's execution within a single tertiary care center with a predominantly Asian demographic underscores concerns regarding the generalizability of our findings. This limitation reflects the broader challenge, as noted by Ishihara et al. [11], of ensuring the universal applicability of clinical findings across varied ethnic and healthcare contexts. Future research endeavors should, therefore, extend to a multicentric approach, engaging a diverse array of populations to enhance the external validity of our results and their applicability across different healthcare systems.

Additionally, our methodology for defining SHGL relied on a blood glucose threshold specific to our cohort, which may not be universally representative. The study's observational design further restricts our ability to infer causality directly, with potential residual confounding factors affecting our outcomes. The absence of serial glucose measurements and detailed hyperglycemia or diabetes management data limited the depth of our analysis, possibly overlooking nuanced metabolic fluctuations.

Furthermore, our research did not address the impact of SHGL on patients with heart failure with preserved ejection fraction (HFpEF), a group that constitutes a significant segment of the heart failure population. Considering the distinct pathophysiological mechanisms and clinical presentations in HFpEF, exploring this area could uncover important insights into SHGL's role across the full spectrum of heart failure syndromes.

By addressing these limitations, future studies can build on our findings, offering a more detailed and universally applicable understanding of SHGL's implications in ADHF management.

## Conclusions

Our study highlights SHGL as a significant, modifiable risk factor that influences mortality, rehospitalization, and the occurrence of MACEs in non-diabetic patients with ADHF undergoing coronary interventions. These findings underscore the importance of integrating SHGL management into comprehensive ADHF care to enhance patient outcomes. Future research should focus on developing standardized SHGL management protocols that accommodate diverse patient demographics to refine heart failure treatment strategies further. This work emphasizes the critical need for proactive metabolic control in improving the prognosis of heart failure patients.
